# DAPK and CIP2A are involved in GAS6/AXL-mediated Schwann cell proliferation in a rat model of bilateral cavernous nerve injury

**DOI:** 10.18632/oncotarget.23978

**Published:** 2018-01-05

**Authors:** Yen-Lin Chen, Yi-Ting Tsai, Ting-Ting Chao, Yi-No Wu, Meng-Chuan Chen, Ying-Hung Lin, Chun-Hou Liao, Shang-Shing P. Chou, Han-Sun Chiang

**Affiliations:** ^1^ Department of Pathology, Cardinal Tien Hospital, New Taipei, Taiwan; ^2^ Department of Chemistry, Fu-Jen Catholic University, New Taipei, Taiwan; ^3^ Graduate Institute of Biomedical and Pharmaceutical Science, Fu-Jen Catholic University, New Taipei, Taiwan; ^4^ Medical Research Center, Cardinal Tien Hospital, New Taipei, Taiwan; ^5^ School of Medicine, Fu-Jen Catholic University, New Taipei, Taiwan; ^6^ Graduate Institute of Medical Sciences, National Defense Medical Center, Taipei, Taiwan; ^7^ Division of Urology, Department of Surgery, Cardinal Tien Hospital, New Taipei, Taiwan

**Keywords:** cavernous nerve, Schwann cell, CIP2A, DAPK, GAS6/AXL

## Abstract

**Purpose:**

Impotence is one of the major complications occurring in prostate cancer patients after radical prostectomy (RP). Self-repair of the injured nerve has been observed in animal models and in patients after RP. However, the downstream signalling is not well documented. Here, we found that the DAPK/CIP2A complex is involved in GAS6/AXL-related Schwann cell proliferation.

**Materials and Methods:**

The 3 groups were a sham group, a 14-day post-bilateral cavernous nerve injury (BCNI) group and a 28-day post-BCNI group. Erectile function was assessed and immunohistochemistry was performed. The rat Schwann cell RSC96 line was chosen for gene knockdown, cell viability, western blot, immunofluorescence and co-immunoprecipitation assays.

**Results:**

The intracavernosal pressure was low on the 14^th^ day after BCNI and partially increased by the 28^th^ day. GAS6 and p-AXL expression gradually increased in the cavernous nerve after BCNI. RSC96 cells incubated with a GAS6 ligand showed increased levels of p-ERK1/2 and p-AKT. Moreover, DAPK and CIP2A.p-AXL and p-DAPK and CIP2A complexes were identified by both immunoblotting and co-immunoprecipitation.

**Conclusion:**

The DAPK/CIP2A complex is involved in GAS6/AXL-related Schwann cell proliferation. CIP2A inhibits PP2A activity, which results in p-DAPK(S308) maintenance and promotes Schwann cell proliferation. CIP2A is a potential target for the treatment of nerve injury after RP.

## INTRODUCTION

The incidence of prostate cancer has consistently increased and is still increasing globally [1, 2]. Radical prostatectomy surgery is the gold standard treatment for localized prostate cancer; however, erectile dysfunction is a major complication of the procedure [3–8]. The regeneration of nNOS (neuronal nitric oxide synthase)-containing nerve fibres is one of the main parameters for the restoration of erectile function. In our previous study, spontaneous nerve recovery could occur as early as 28 days after bilateral cavernous nerve injury (BCNI) in a rat model, as determined via electron microscopy examination. The regeneration of Schwann cell myelin sheaths was especially prominent on the 28^th^ day after BCNI; more Ki-67-positive Schwann cells were present. This finding was consistent with the transmission electron microscope findings that revealed the presence of regeneration on the 28^th^ day. In the peripheral nervous system, Schwann cells promote nerve regeneration by secreting trophic support molecules and establishing a supportive growth matrix. The identification of growth factors that promote Schwann cell outgrowth after nerve injury is an important strategy for improving nerve regeneration [9].

Growth arrest-specific protein 6 (GAS6) is a major vitamin K-dependent, *r*-carboxylated, secreted growth factor that functions in cell survival, adhesion, chemotaxis, mitogenesis, and cell growth [10–12]. GAS6shows higher affinity for its receptor AXL than for Tyro3 and Mer, and it is often called the GAS6/AXL pathway [13–15]. AXL, a receptor tyrosine kinase, is broadly expressed and its onset of expression occurs in late embryogenesis [16]. AXL was initially discovered in cancer cells more than two decades ago [17]. The GAS6/AXL pathway regulates various functions in the cell, including survival, growth, aggregation, migration and anti-inflammation [18]. There is also increasing evidence to indicate that overexpression and elevation of AXL activity is related to chronic pathological disorders [19]. Activation of AXL is also implicated in the progression of cardiovascular diseases [20, 21]. Moreover, Li et al. [22] demonstrated that tyrosine phosphorylation of Ax1 after incubation with GAS6 can promote Schwann cells growth via extracellular signal-regulated kinase (ERK) signalling.

Death-associated protein kinase (DAPK) is a serine/threonine kinase that is involved in signal transduction during central nervous system development. In the chick neural tube, neogenin can interact with DAPK, reduce DAPK autophosphorylation on Ser308 and lead to neuronal cell death [23]. In acute models of injury such as ischaemia and seizure, DAPK is elevated in injured neurons [24]. Protein phosphatase 2A (PP2A) is a ubiquitous serine/threonine protein phosphatase that regulates many biological processes. Elevated PP2A mRNA expression was found in an ischaemia rat model [25]. However, the role of PP2A in neuron regeneration is still unknown. In addition, cancerous inhibitor of protein phosphatase 2A (CIP2A) is an endogenous cellular inhibitor of PP2A. It has been reported that the maintenance of phosphorylated DAPK (S308) via the inhibition of PP2A may sustain cell survival in the presence of neutrin-1 [26].

The present study is designed to examine whether GAS6/AXL-induced Schwann cell proliferation can contribute to autonomous recovery from BCNI in a rat model and *in vitro*. In this study, both DAPK and CIP2A were found to be involved in GAS6/AXL-related Schwann cell proliferation. CIP2A inhibits PP2A activity, resulting in p-DAPK (S308) maintenance and the promotion of Schwann cell proliferation. CIP2A is a potential target for the treatment of nerve injury after radical prostatectomy.

## RESULTS

### GAS6 and p-AXL are expressed in the regenerative cavernous nerve in a rat model of BCNI

**Figure 1:**
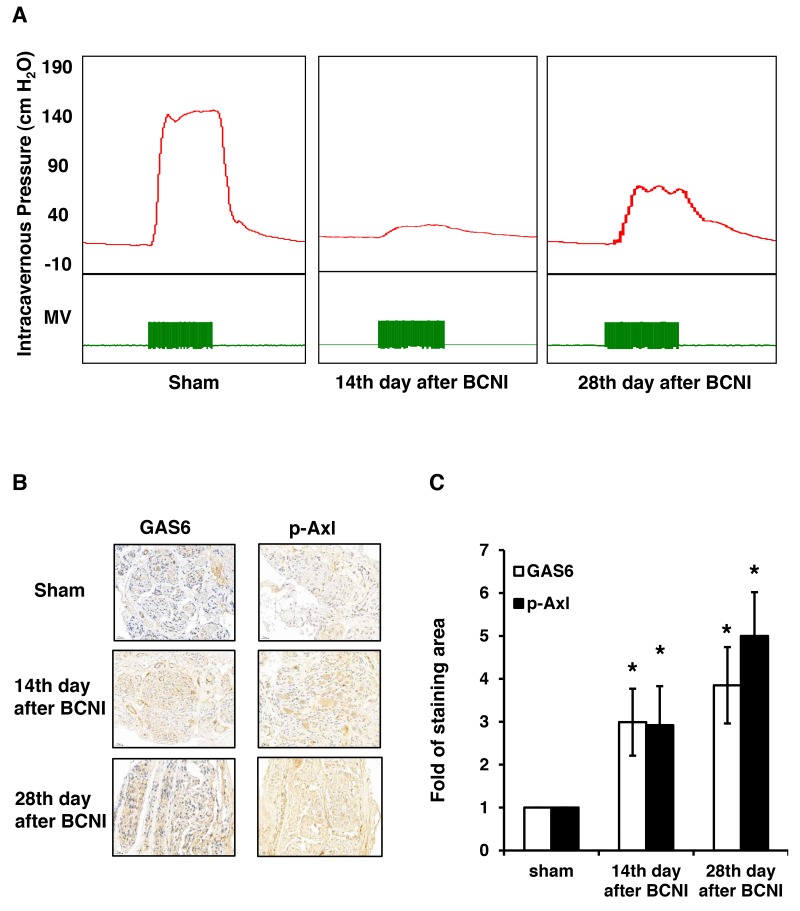
GAS6 and p-AXL are expressed in the regenerative cavernous nerve of rats subjected to BCNI **(A)** Representative ICP tracing responses of each group (n=5 per group) to the stimulation of the cavernous nerve at 14 days and 28 days. The green bar represents an electrical stimulus duration of 60 seconds. **(B)** Immunohistochemistry images demonstrated that GAS6 and p-AXL expression levels increased in the cavernous nerve after BCNI. **(C)** The quantitative analysis results showed a significantly higher expression of GAS6 and p-AXL in the cavernous nerve of animals subjected to injury after 28 days.

The ICP was lowest on the 14^th^ day after BCNI and partially increased on the 28^th^ day, which was similar to our previous results (Figure 1A). Therefore, we determined whether GAS6 and p-AXL were expressed in the BCN injured rats. The GAS6 and p-AXL expression levels gradually increased in the cavernous nerve after BCNI (Figure 1B). The staining pattern was mainly in the cytoplasm, with focal staining on the 14^th^ day and more diffuse staining on the 28^th^ day. The quantitation results showed that the expression of both GAS6 and p-AXL significantly increased in the cavernous nerve after injury (Figure 1C).

### GAS6 induced Schwann cell proliferation

**Figure 2:**
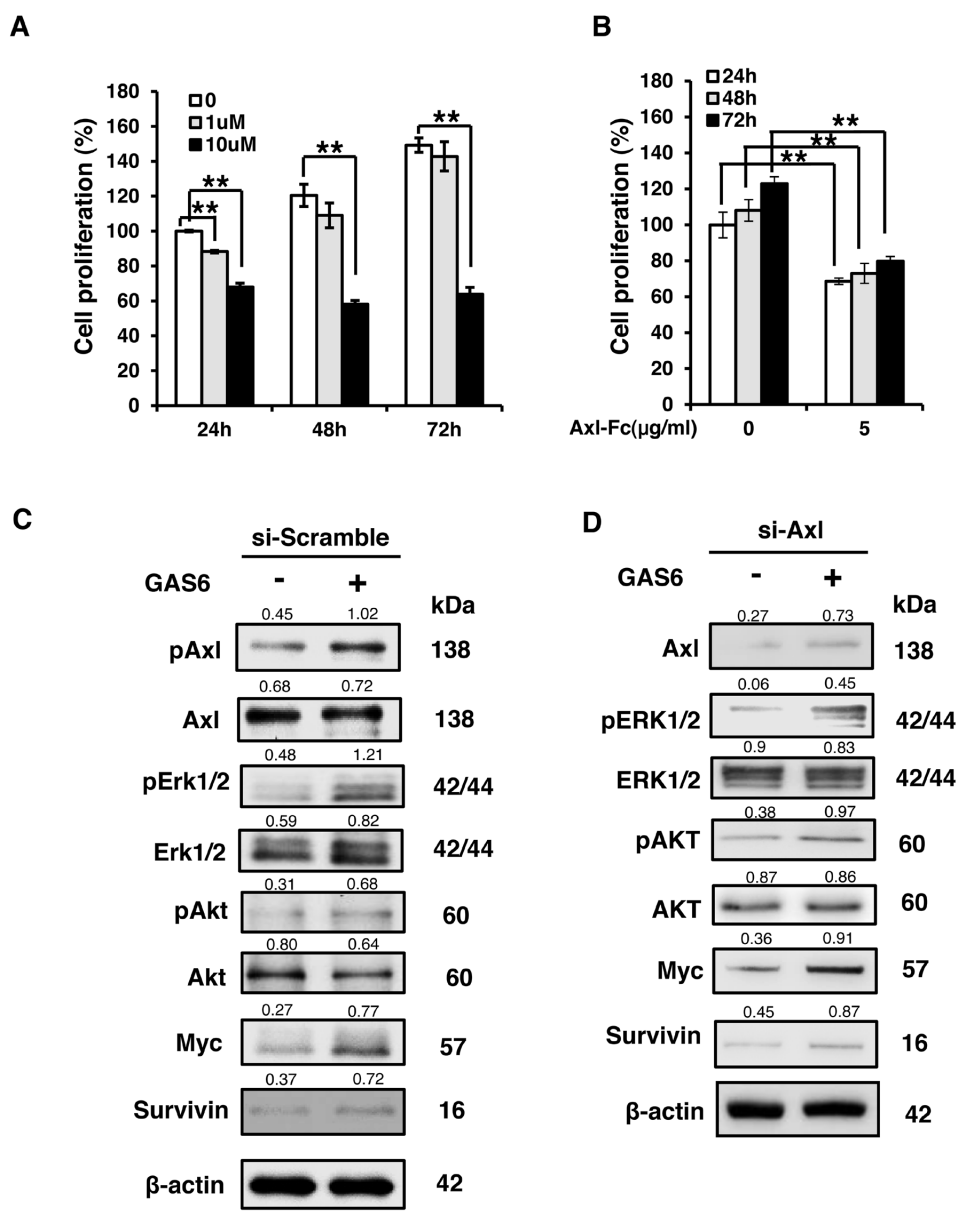
GAS6 induces Schwann cell proliferation **(A)** Time- and dose-dependent effects of LDC1267 in RSC96 cells. **(B)** Time- and dose-dependent effects of AXL-Fc in RSC96 cells. The inhibitory effects of cell proliferation were increased in a time-dependent manner (31.3%, 35.1% and 43.1% decreased in 24 h, 48 h, and 72 h, respectively). Data are the mean ± SD, and n = 3 for each concentration and time point. ^*^ p < 0.05, ^**^ p < 0.01, vs. control. **(C)** RSC96 incubated with GAS6 (100 ng/ml) for 30 min and immunoblotting evaluations of pAxl, Axl, pERK1/2, ERK1/2, pAKT, AKT, Myc and Survivin. **(D)** RSC96 cells were transfected with either control or Axl siRNA for 48 hours and then exposed to GAS6 (100 ng/ml) for 30 min. The relative quantification of protein expression was normalized with respect to β-actin expression.

To investigate the GAS6/AXL signalling pathway in nerve injury, the Schwann cell line RSC96 was used in this study. LDC1267 is an AXL inhibitor that can block the GAS6/AXL pathway. The percentage of cell proliferation was dose-dependently decreased in the presence of LDC1267 at the indicated times (Figure 2A). As LDC1267 is a pan-TAM inhibitor, the possible toxicities of RSC96 cells may be a concern and bias the experimental results. Therefore, we used a soluble form of AXL, the AXL-Fc protein, to neutralize GAS6. AXL-Fc is an alternative method by which to inhibit the GAS6/AXL pathway by blocking GAS6. The results indicated that the proliferation rate decreased when cells were treated with AXL-Fc (Figure 2B). The inhibitory effect of cell proliferation was increased in a time-dependent manner (31.3%, 35.1% and 43.1% decreased after 24 h, 48 h, and 72 h, respectively). Next, we examined the GAS6/AXL downstream proteins by western blotting. The result showed that GAS6 induces downstream signals of ERK1/2 and AKT activation and increases the expression of MYC and Survivin through GAS6/AXL (Figure 2C). When we knocked down AXL, downstream signalling was inhibited after treatment with GAS6 (Figure 2D). However, the GAS6-induced downstream signal was mainly, but not completely, mediated through AXL. This effect occurs because GAS6 not only binds to AXL but also binds to Tyro3 and Mer [14, 15]. These results indicate that GAS6 can influence Schwann cell growth through AXL signalling.

### GAS6 increased the expression of both CIP2A and p-DAPK

**Figure 3:**
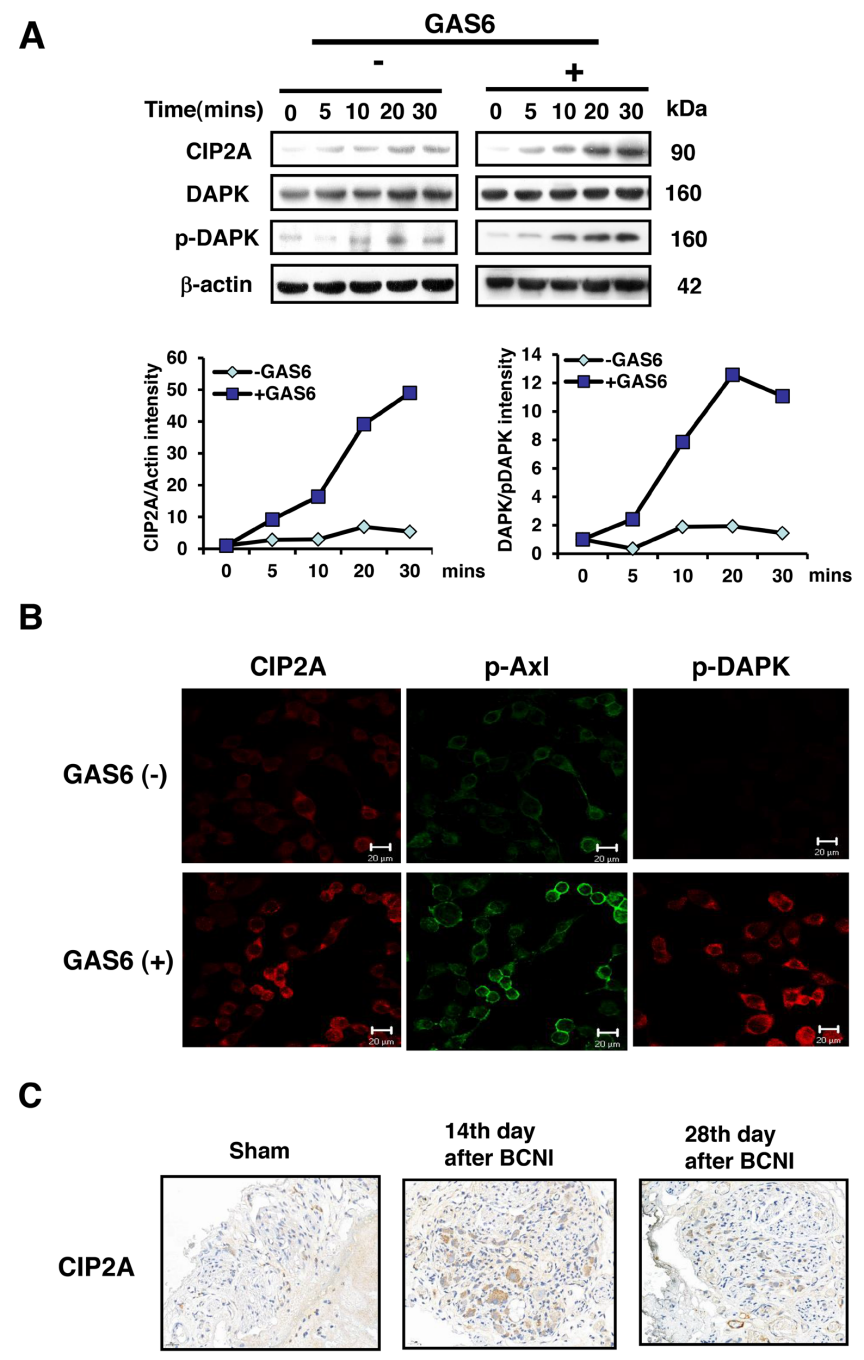
GAS6 increases the amount of both CIP2A and p-DAPK in RSC96 cells **(A)** RSC96 cells were incubated with GAS6 (100 ng/ml), and immunoblotting evaluations of CIP2A and p-DAPK expression were performed. The lower panel shows the intensity during basal conditions from the scanned imaged (upper panel). **(B)** RSC96 cells incubated with and without GAS6 (100 ng/ml) for 30 min and analysed by immunofluorescence. **(C)** Immunohistochemistry images demonstrated that CIP2A expression levels were higher than sham levels at 14 days and 28 days in the cavernous nerve after BCNI.

To further elucidate the molecular mechanism of the GAS6/AXL pathway involved in Schwann cell proliferation, RSC96 cells were incubated with GAS6 to mimic the microenvironment of nerve injury *in vitro*. The results showed that the protein expression of CIP2A and p-DAPK increased when GAS6 was present, as detected by both immunoblotting (Figure 3A) and immunofluorescence (Figure 3B) methods. Both p-DAPK and CIP2A were significantly increased in a time-dependent manner in the presence of GAS6. Moreover, CIP2A, p-DAPK and p-AXL were all expressed in the cytoplasm of the RSC96 cells. CIP2A expression was also observed in the BCN injured rats; the expression increased by the greatest amount on the 14^th^ day and was reduced by the 28^th^ day after BCNI compared with the sham group (Figure 3C). Therefore, CIP2A may be involved in the early stage, but not the late stage, of nerve injury in the BCNI rat model.

### GAS6 triggers p-AXL, p-DAPK and CIP2A to form a protein complex

**Figure 4:**
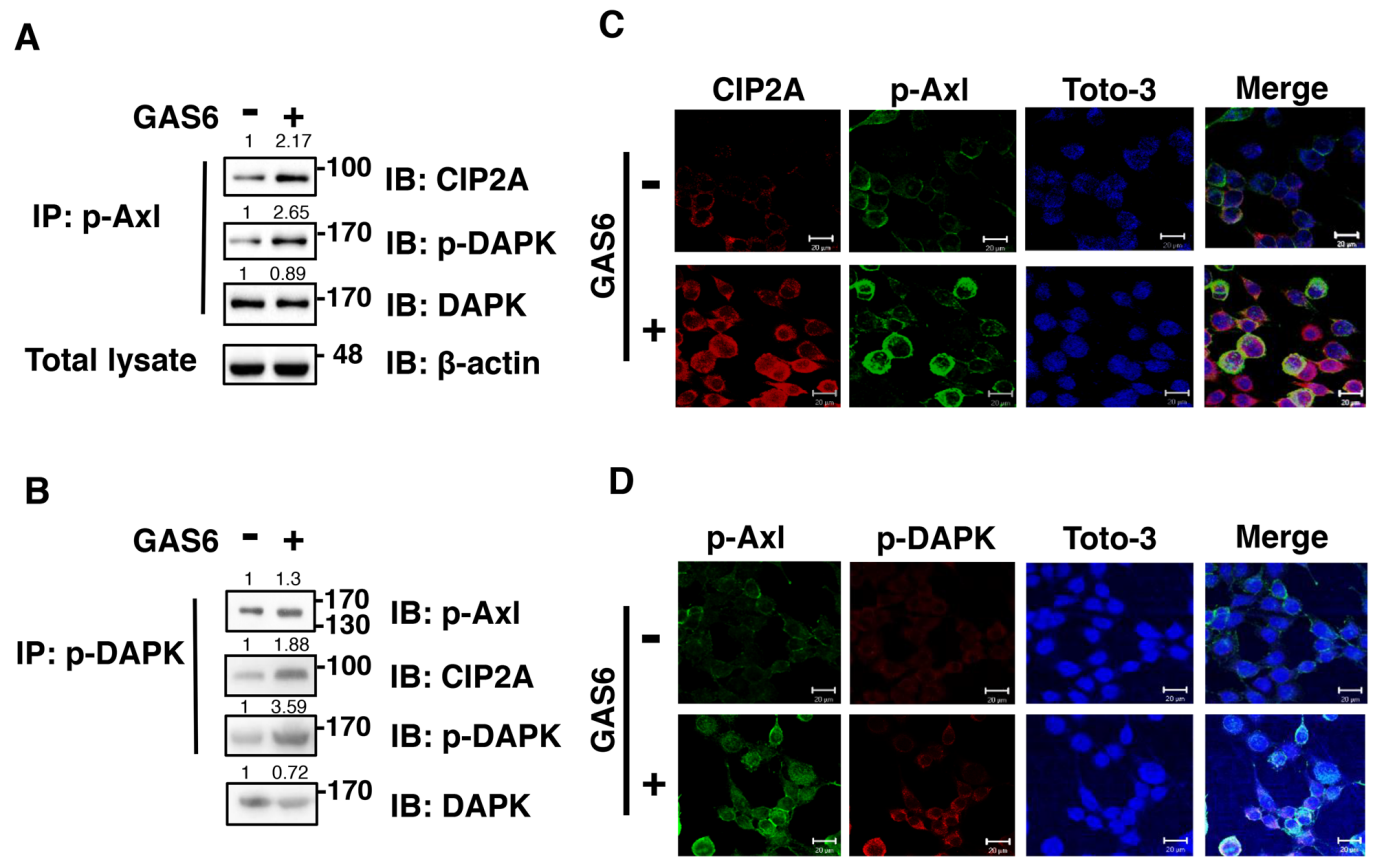
GAS6 triggers p-Axl, p-DAPK and CIP2A to form a protein complex in RSC96 cells **(A-B)** Co-immunoprecipitation between p-Axl, p-DAPK and CIP2A with GAS6 stimulation for 30 min in RSC96 cells. Total lysate indicates 1/10 input in each experiment. The relative quantification of protein expression was normalized with respect to β-actin expression. **(C)** Immunofluorescence image demonstrating co-localized CIP2A and p-Axl and co-localized p-Axl and p-DAPK **(D)** after GAS6 stimulation for 30 min in RSC96 cells.

To investigate the relationships among p-AXL, p-DAPK and CIP2A, we performed immunoprecipitation experiments. Equal concentrations of p-AXL were used for immunoprecipitation, and both endogenous p-DAPK and CIP2A were detected in control and GAS6-induced cells (Figure 4A). Co-localized CIP2A and p-AXL in the cytoplasm of RSC96 Schwann cells was also observed by immunofluorescence analysis (Figure 4B). To further confirm that p-AXL, p-DAPK and CIP2A form a complex, an equal concentration of DAPK was used for immunoprecipitation. Similar results were observed, whereby endogenous p-AXL, p-DAPK and CIP2A were detected in control and GAS6-induced cells (Figure 4C). p-AXL and p-DAPK were also co-localized in the cytoplasm of RSC96 Schwann cells (Figure 4D). In summary, these results showed that p-AXL, p-DAPK and CIP2A form a protein complex during GAS6-AXL signalling.

### GAS6 triggered Schwann cell proliferation mainly through CIP2A and DAPK

**Figure 5:**
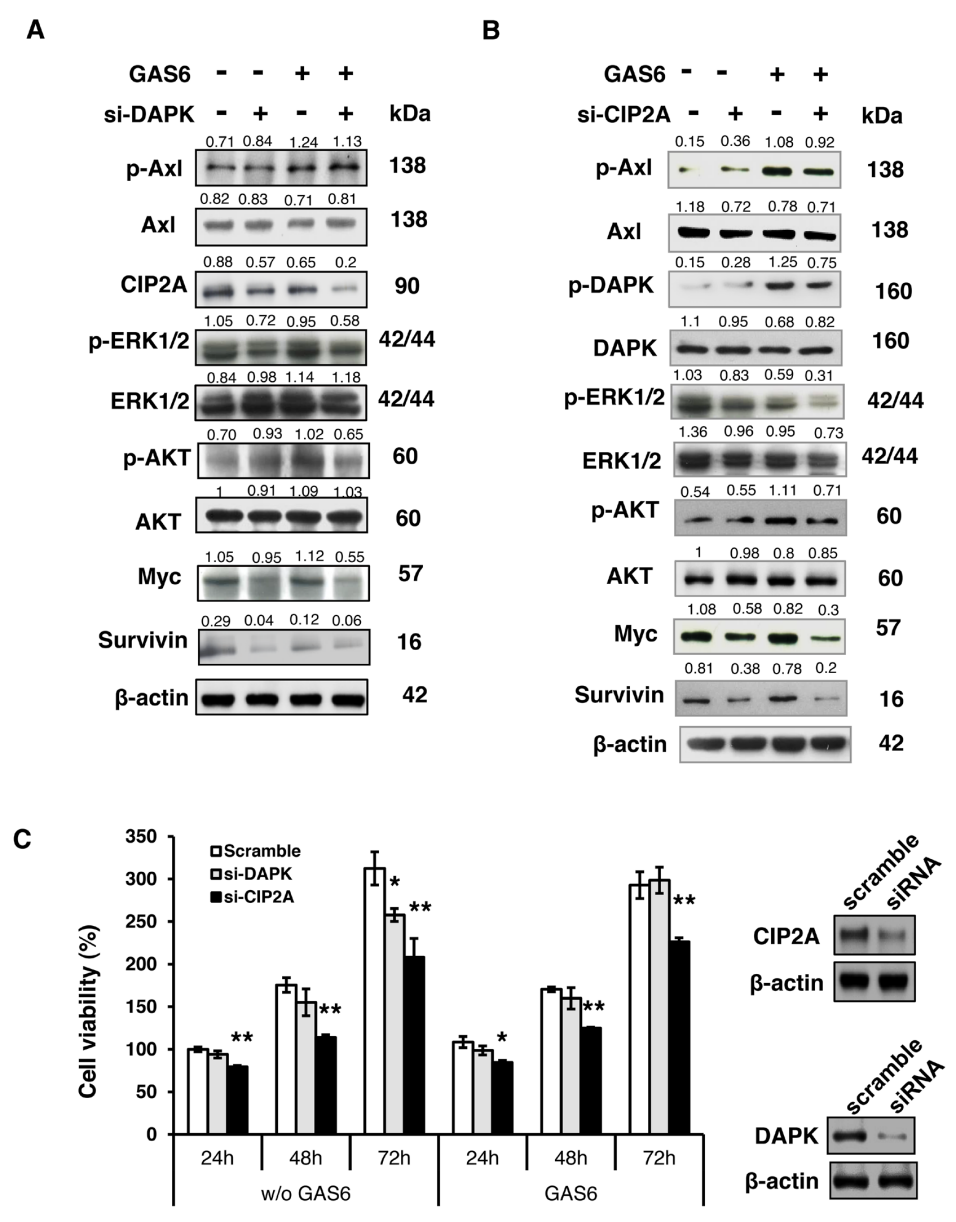
GAS6 triggered Schwann cell proliferation primarily through CIP2A and DAPK **(A)** RSC96 cells were transfected with either control or DAPK siRNA for 48 h and then exposed to GAS6 (100 ng/ml) for 30 min. Immunoblotting evaluations of pAxl, Axl, CIP2A, DAPK, pERK1/2, ERK1/2, pAKT, AKT, Myc and Survivin. **(B)** RSC96 cells were transfected with either control or CIP2A siRNA for 48 h and then exposed to GAS6 (100 ng/ml) for 30 min. Immunoblotting evaluations of pAxl, Axl, DAPK, pDAPK, pERK1/2, ERK1/2, pAKT, AKT, Myc and Survivin. **(C)** DAPK or CIP2A was knocked down for 48 h then RSC96 cells were incubated with GAS6 (100 ng/ml) at the indicated hours. Cell viability was analysed via the WST-1 assay. Data are the mean ± SD, and n = 3 for each time point. ^*^ p < 0.05, ^**^ p < 0.01 vs. scramble.

To confirm that Schwann cell proliferation elicited by GAS6 triggered complex formation (p-AXL, p-DAPK and CIP2A), DAPK and CIP2A knockdown experiments were performed. The downstream signals of p-ERK1/2 and p-AKT were induced by GAS6 but inhibited by DAPK knockdown. The expression of other proliferation signals, such as MYC and Survivin, was also decreased when DAPK was knocked down (Figure 5A). Similar results were observed with the knockdown of CIP2A, which was another protein complex component (Figure 5B). Furthermore, the results of the WST-1 functional proliferation assay showed decreasing cell viability when DAPK or CIP2A were knocked down compared with cells transfected with scramble siRNA (Figure 5C). The inhibitory effect of si-CIP2A was higher than that of si-DAPK in terms of the proliferation rate probably because PP2A activity will be altered when CIP2A is knocked down. CIP2A had been described as an endogenous PP2A inhibitor. Once PP2A was activated, it could dephosphorylate several proteins, such as p-ERK and p-AKT, which would inhibit cell growth.

### CIP2A inhibited PP2A-mediated DAPK dephosphorylation

**Figure 6:**
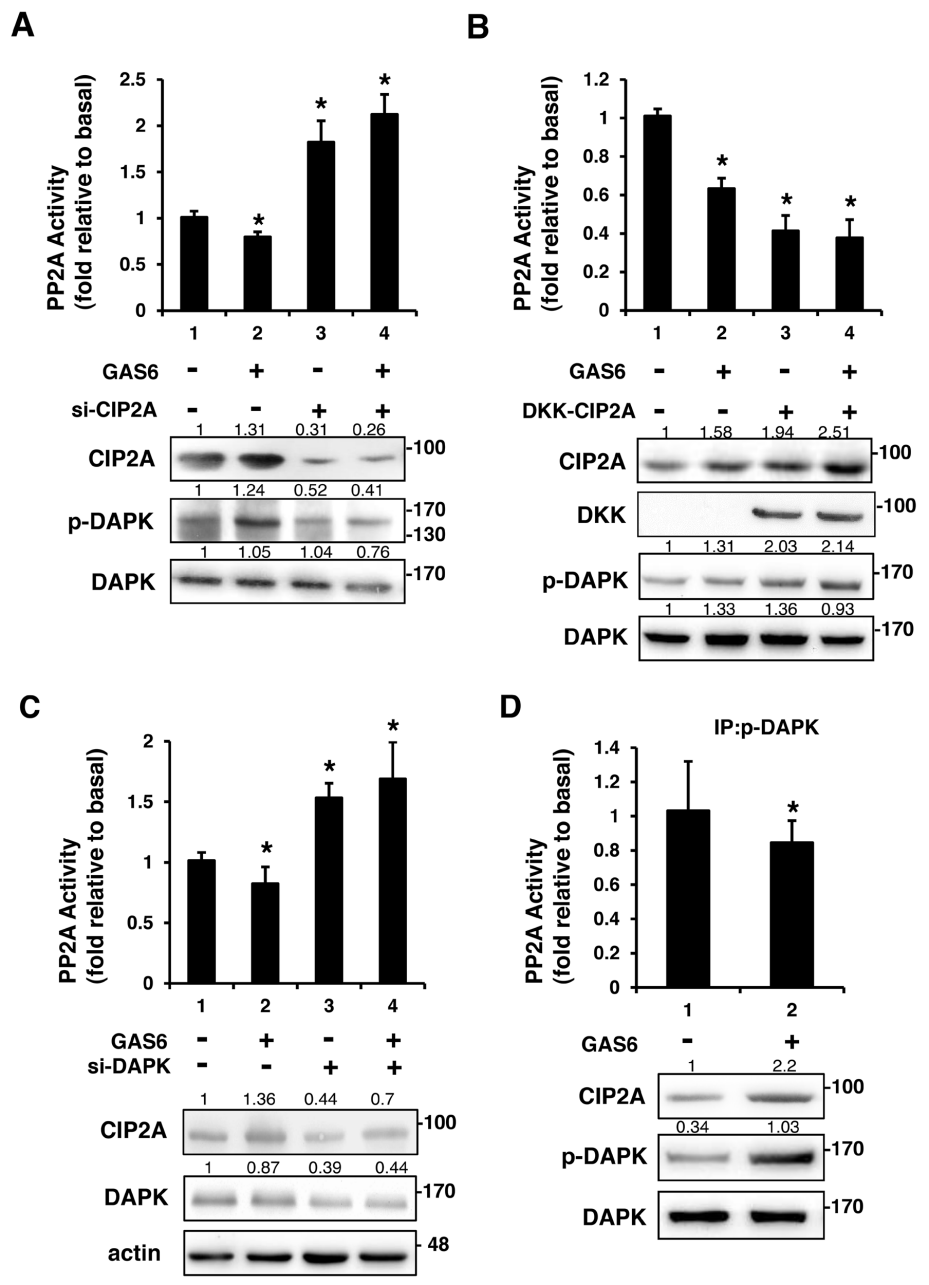
CIP2A is an inhibitor of PP2A-mediated DAPK dephosphorylation RSC96 cells were transfected with control, CIP2A siRNA **(A)**, DKK-CIP2A **(B)**, or DAPK siRNA **(C)** for 48 h and then exposed to GAS6 (100 ng/ml) for 30 min. PP2A activity and immunoblotting evaluations of DKK, CIP2A, pDAPK, and DAPK were performed. **(D)** RSC96 cells were incubated with GAS6 (100 ng/ml) for 30 min and co-immunoprecipitation between CIP2A, pDAPK and PP2A was evaluated. Immunoprecipitated PP2A activity was measured. Data are the mean ± SD, and n = 3 for each time point. ^*^ p < 0.05, ^**^ p < 0.01, vs. control.

**Figure 7:**
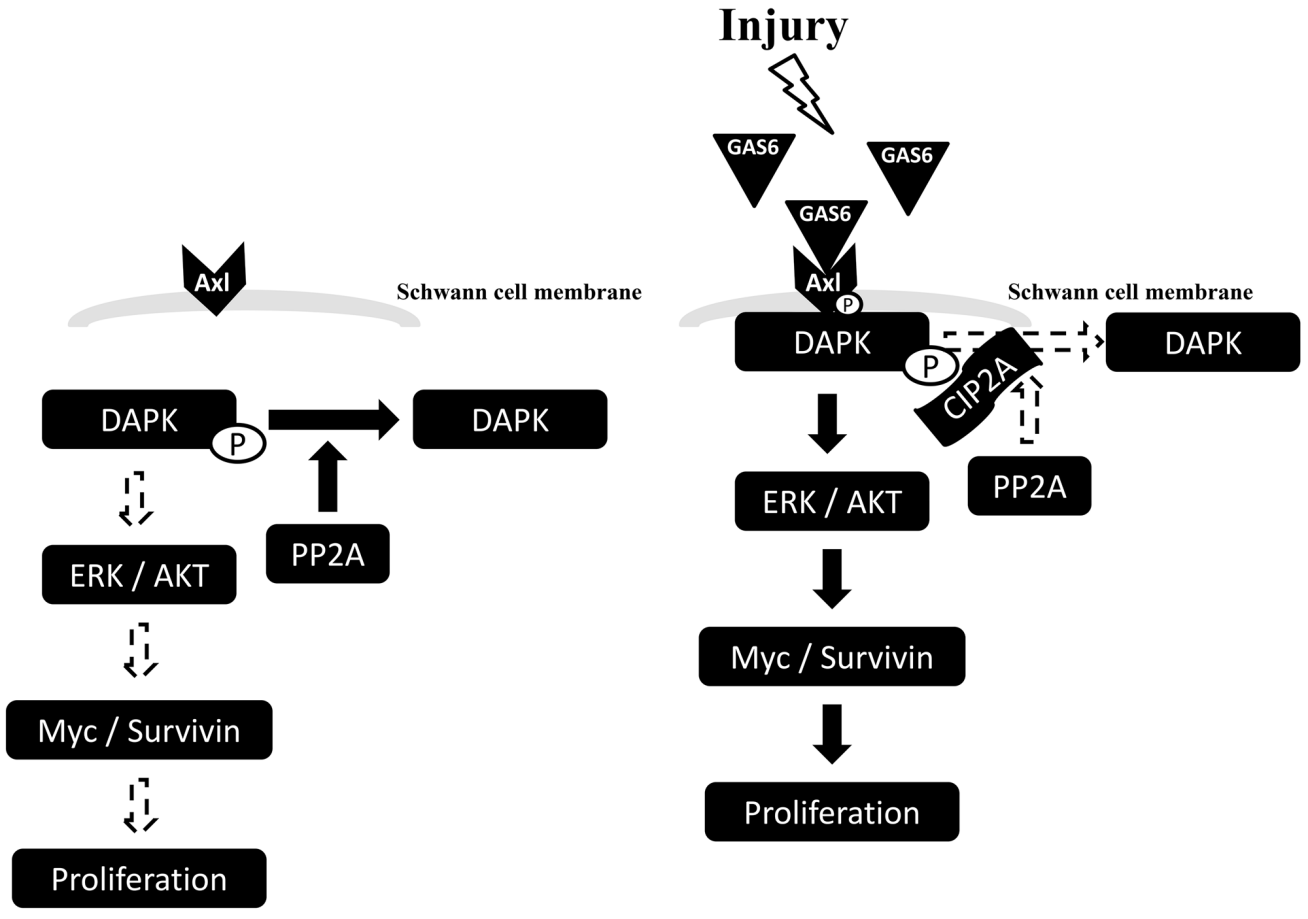
Schematic diagram of GAS6-induced Schwann cell proliferation GAS6 binds to its receptor, AXL, and p-AXL induce Schwann cell proliferation via MYC and Survivin signalling. GAS6 increases the expression level of CIP2A to form a p-AXL, p-DAPK and CIP2A protein complex. The presence of CIP2A inhibits the enzyme activity of PP2A and activates downstream ERK1/2 and AKT signals for proliferation.

To further elucidate the role of PP2A in the GAS6-triggered p-AXL, p-DAPK and CIP2A protein complex formation, PP2A activity was measured. PP2A activity decreased in the presence of GAS6. However, PP2A activity increased with the knockdown of CIP2A (Figure 6A). By contrast, overexpression of CIP2A decreased PP2A activity regardless of the presence of GAS6. However, PP2A activity was still higher in the presence of GAS6 when CIP2A was overexpressed (Figure 6B). A similar result was seen with the knockdown of DAPK. PP2A activity increased with the knockdown of DAPK (Figure 6C). PP2A activity was lowered by immunoprecipitated p-DAPK in the presence of GAS6 (Figure 6D). These findings indicate that GAS6-induced Schwann cell proliferation occurs through p-DAPK, and the inhibition of PP2A dephosphorylates p-DAPK by CIP2A. GAS6 can increase the expression of CIP2A to form the p-AXL, p-DAPK and CIP2A protein complex. The presence of CIP2A will inhibit PP2A activity and activate the downstream ERK1/2 and AKT signals for proliferation (Figure 7).

## DISCUSSION

The present study investigated the possible underlying molecular signalling of Schwann cells in a rat model of BCNI. The first finding is that the expression levels of GAS6 and AXL were increased when cavernous nerve injury was induced in rats. An *in vitro* study showed that GAS6/AXL mediated Schwann cell proliferation via p-DAPK. Furthermore, GAS6-induced AXL signalling can increase the expression of CIP2A and facilitate the formation of a complex of p-AXL, p-DAPK and CIP2A. The presence of CIP2A inhibited the enzyme activity of PP2A and activated the downstream ERK1/2 and AKT signals for proliferation. Therefore, this study is the first to demonstrate that the partial recovery of erectile function after BCNI involves GAS6/AXL-mediated Schwann cell proliferation via the formation of a p-AXL, p-DAPK and CIP2A protein complex to regulate downstream ERK1/2 and AKT signalling.

In patients who have undergone radical prostatectomy surgery, complete or partial recovery of erectile function can occur without any specific treatment. Therefore, spontaneous regeneration of the injured nerve aroused researchers’ attention. Zhang *et al.* have reported that in rats subjected to unilateral CNS ablation, nNOS-containing nerve fibres would regenerate 6 months later. Our previous study found that the regeneration process might start at the 14^th^ day after injury. The regeneration of the Schwann cell myelin sheath could be completed on the 28^th^ day after nerve injury. GAS6 and p-AXL expression was detected in the BCNI rat via immunohistochemistry, and expression was prominent on the 14^th^ and 28^th^ days when compared with the sham group. GAS6 is a soluble ligand that is reported to have neuroprotective properties. The delivery of GAS6 directly into an animal model of autoimmune encephalomyelitis preserved central nervous system function [10]. Moreover, tyrosine phosphorylation of AXL after incubation with GAS6 can promote Schwann cell growth via ERK2 signalling [22]. GAS6 can activate Tyro3, AXL and Mer family proteins; however, AXL showed the highest affinity for GAS6 [12]. In our study, we detected GAS6 and p-AXL in the cavernous nerve via immunohistochemical staining to demonstrate that GAS6-AXL signalling had been activated in the BCNI rat model. When we tried to identify the possible molecule involved in the downstream signalling, we first focused on the ERK pathway. ERK kinase binds to the DAPK death domain and activates its catalytic activity through phosphorylation [27]. Therefore, we hypothesized that DAPK may be involved in the downstream signalling cascade. DAPK is a crucial intracellular protein that mediates cell death induction through a wide spectrum of apoptotic and non-apoptotic signals via its serine-threonine kinase activity [28–31]. Although DAPK is a key player in cell death regulation, very few direct activators of its catalytic activity have been identified. For instance, calmodulin binds to the calmodulin regulatory region of DAPK and is activated by a calcium spike [32]. We have demonstrated that GAS6-induced AXL signalling can maintain the phosphorylated form of DAPK and activate the downstream ERK1/2 and AKT signals for proliferation.

PP2A is a ubiquitously expressed serine-threonine phosphatase that accounts for a large fraction of phosphatase activity in eukaryotic cells. However, PP2A is not a single molecule but rather a collection of oligomeric enzymes, each of which contains a common catalytic subunit, PP2Ac. This catalytic subunit interacts with the PR65 scaffold protein and with a wide family of regulatory B subunits [33]. Two alternative genes, PR65a and PR65b, encode two forms of the PR65 scaffold protein. Although most PP2A holoenzymes contain the PR65a isoform, PR65b appears to play a key regulatory role in cancer. Indeed, PR65b is decreased or mutated in a large fraction of human cancers and has recently been causally linked to cancer development [34]. Furthermore, PP2A inactivation in cancer also occurs frequently through the upregulation of CIP2A, a PR65 interactor and PP2A inhibitor [35]. Here, we showed that p-AXL, p-DAPK, and CIP2A form a dynamic protein complex that regulates GAS6-mediated Schwann cell proliferation. Initially, CIP2A was identified as an oncogene in gastric and liver cancer. Protein kinase B (PKB/AKT) and UNCEB5 are only two of the CIP2A substrates that have been identified. CIP2A enhanced AKT-mediated cell survival through the direct regulation of AKT-associated PP2A phosphatase activity. In addition, CIP2A controls cell-cycle progression, and depletion of CIP2A results in extended cell division [36]. These results indicated that CIP2A has a broader role in regulating cell proliferation, as previously expected [37]. The current study is the first to demonstrate that CIP2A is involved in GAS6-mediated Schwann cell proliferation.

In conclusion, DAPK and CIP2A are involved in GAS6/AXL-related Schwann cell proliferation. CIP2A inhibits PP2A activity, which results in p-DAPK (S308) maintenance and promotes Schwann cell proliferation. CIP2A is a potential target for the treatment of nerve injury after radical prostatectomy.

## MATERIALS AND METHODS

### Experimental animals

Fifteen 12-week-old male Sprague-Dawley rats (weight, 450–600 g) were used in this study. Five rats were in each group. All animals were supplied by BioLasco Taiwan Co., Ltd. (Taipei, Taiwan), and all experiments were approved by the Fu Jen Catholic University Animal Care and Use Committee (IACUC approval NO: A10320). All study procedures and methods were carried out in accordance with the approved guidelines.

### Experimental design

The animals were randomly assigned to the following 3 groups: a sham group, a 14-day post-BCNI group and a 28-day post-BCNI group. At the end of the 28^th^ day in the sham group and each specific day in the BCNI groups, the erectile response was measured. In addition, H&E staining and immunohistochemical staining were performed on histological sections of the cavernous nerve.

### Surgical procedures

For the surgical procedure, the animals were first anaesthetized with an intraperitoneal injection of sodium pentobarbital (40 mg/kg). After the abdomen was shaved and wiped with an iodine-based solution, a lower midline abdominal incision was made. The prostate gland was exposed, and the posterolateral cavernous nerve and the major pelvic ganglion were identified. Apart from the sham group, all rats were subjected to BCNI. In the sham group, no further surgery was carried out and the abdomen was closed. In the other groups, the cavernous nerve was isolated and a crush injury was established with a haemostat clamp (Roboz Surgical Instrument Co. Inc., Gaithersburg, USA) for 2 minutes before closing the abdomen.

### Measurement of erectile responses

The cavernous nerve was exposed and isolated via a repeat midline abdominal incision, and the crura of the penis were identified. A 24-G needle containing 50 U/mL of heparin solution was inserted into the right penile crus and connected to polyethylene-50 tubing for measuring the intracavernous pressure (ICP) using an MP36 pressure transducer (Biopac Systems Inc., CA, USA) and BSL 3.7.3 software. The cavernous nerve was stimulated using a bipolar stainless steel electrode. Monophasic rectangular pulses were generated by a computer with a DS3 constant current isolated stimulator (AutoMate Scientific Inc., CA, USA). The stimulus parameters included a 7.5 mA amplitude, a 20 Hz frequency, a 0.2 ms pulse width, and a 60 s duration. A real-time response of the erectile tissue was determined based on the maximal ICP, the changes in ICP (ΔICP), and the area under the ICP curve.

### Histology and immunohistochemistry

Tissue paraffin-embedded blocks were constructed and cut in 5-μm thick sections for H&E staining. Immunohistochemical stains were performed using a Ventana BenchMark XT automated stainer (Ventana, Tucson, AZ). Primary antibodies against GAS6 (1:100, TA323562; Origene), p-AXL (Y779) (1:100, AF2228, R&D), and CIP2A (1:50, NB100-74663, Novus) were used. A Ventana OptiView DAB detection kit was used, and the slides were counterstained with haematoxylin, dehydrated, and mounted.

### Cell culture

The rat Schwann cell line RSC96 was used in this study. The cell line was obtained from the American Type Culture Collection (Manassas, VA). The RSC96 cell line was maintained in DMEM with 4 mM L-glutamine adjusted to contain 1.5 g/L sodium bicarbonate, 4.5 g/L glucose and FBS at a 9:1 ratio in a 37 °C humidified incubator with 5% CO_2_ in air.

### Reagents and antibodies

AXL-Fc protein fragment (AXL-Fc) was purchased from R&D Systems (St. Paul, MN, USA). LDC1267 (a TAM family inhibitor) was purchased from Selleck (Houston, TX, USA). Rat GAS6 recombinant protein (RPA204Ra01) was purchased from Cloud-clone Corp. Antibodies for immunoblotting, including DAPK, p-DAPK (S308), CIP2A, AKT, MYC, Survivin and Actin, were purchased from Santa Cruz Biotechnology (San Diego, CA). ERK1/2, p-ERK1 (T202/Y204) / ERK2 (T185/Y187), and p-AXL (Y779) were purchased from R&D Systems (St. Paul, MN, USA). Other antibodies, including anti-PP2A and p-AKT (Ser473), were purchased from Cell Signaling (Danvers, MA).

### Cell proliferation assay

RSC96 cells were seeded in 96-well plates (2.5 × 10^3^ cells/well), and 10% WST-1 agent (water-soluble tetrazolium monosodium salt) (Cell Proliferation Reagent WST-1; Roche Applied Science, Indianapolis, IN) was added to the cell suspension in each well. Cells were then incubated for 1-2 hours, and cell proliferation was quantified by measuring the absorbance at 450 nm using a Biotek Synergy HT ELISA reader (Biotek, Winooski, VT).

### Immunofluorescence for CIP2A, p-AXL and p-DAPK Localization in RSC96 cells

Cells treated with or without GAS6 were plated on a glass slide and fixed in 4% paraformaldehyde and 0.2% Tween 20 in PBS. After a brief wash in PBS, the slides were blocked with 5% normal serum in 1% BSA/0.2% Triton X-100 for 1 h and incubated with mouse monoclonal anti-CIP2A antibody (1:50; Santa Cruz), rabbit polyclonal anti-p-AXL antibody (1:40; R & D) and mouse polyclonal anti-p-DAPK antibody (1:50; Biorbyt). After an overnight incubation, the slides were washed and then incubated with fluorescent (Alexa 568, Alexa 488) conjugated secondary antibodies (1:200) for 1 h and counterstained for nuclei with ToTo-3. The stained slides were mounted and analysed by epifluorescence microscopy (Leica). Pictures were captured using a Photometrics CoolSNAP EZ system (high-performance EMCCD and CCD Cameras) and MetaMorph version software (Molecular Devices).

### Co-immunoprecipitation and western blot analyses

For the immunoprecipitation of endogenous p-DAPK or p-AXL from cells, cells were lysed at 4 °C in modified RIPA buffer (50 mM Tris-HCl, pH 7.8, 150 mM NaCl, 5 mM EDTA, 0.5% Triton X 100, 0.5% Nonidet-P40, and 0.1% sodium deoxycholate) and a protease inhibitor mixture (Complete, Roche Molecular Biochemicals) and were subjected to immunoprecipitation with the indicated antibodies. Lysates containing comparable amounts of protein, as estimated with a BCA protein assay (Thermo Fisher Scientific, Waltham, MA), were subjected to western blotting. For immunoprecipitation, 1 mg of protein-containing lysate was incubated with the appropriate antibody for 3 h or overnight and then with protein A/G-magnetic beads overnight at 4 °C. Antigen-antibody-bead complexes were centrifuged, washed, resuspended in the sample buffer (60 mM Tris-Cl, pH 6.8, 2% SDS, 10% glycerol, 5%-mercaptoethanol, and 0.01% bromphenol blue), denatured, subjected to SDS-PAGE, transferred to nitrocellulose membranes, and probed with primary antibodies followed by secondary antibodies coupled to horseradish peroxidase.

### Ectopic expression of CIP2A

CIP2A cDNA (KIAA1524) was purchased from Origene (Rockville, MD). Cells were transfected with the CIP2A construct, which was carried out using X-tremeGENE HP Transfection Reagent (Roche, Diagnostics Corp, IN) according to the manufacturer’s instructions. After 48 hours of transfection, RSC96 cells were treated with GAS6 for an additional 30 mins and then harvested for western blot and PP2A activity analyses.

### PP2A phosphatase activity

PP2A activity was measured in fresh cells, as described previously, using an R&D Systems PP2A DuoSet IC activity assay kit according to the manufacturer’s instructions (R&D Systems, Minneapolis, MN). Briefly, an immobilized capture antibody specific for the catalytic subunit of PP2A that binds both active and inactive PP2A was used. After washing, a substrate was added that is dephosphorylated by active PP2A to generate free phosphate, which is then detected by a sensitive dye-binding assay using malachite green and molybdic acid.

### Gene knockdown using siRNA and cell transfection

Control (sc-37007) and DAPK (sc-38977) siRNA were purchased from Santa Cruz Biotechnology (San Diego, CA). CIP2A (4390771) was purchased from Thermo Fisher Scientific Inc. (Waltham, MA). Cells were transfected with siRNA to a final concentration of 100 nM in 6-well plates with X-tremeGENE siRNA Transfection Reagent (Roche, Diagnostics Corp, IN) according to the manufacturer’s instructions. After 48 hours, the medium was replaced and the cells were harvested for western blot analysis.

### Statistical analysis

All statistical analyses were performed using SPSS 18.0 software (SPSS Inc., Chicago, IL). Densitometric quantification and normalization were performed using ImageJ 1.42q software. The relative quantification of protein expression was normalized with respect to β-actin expression. Data are presented as the mean ± standard deviation. Statistical analysis was performed using a two-tailed Student’s *t*-test. The results are expressed as the mean ± standard deviation (SD). Differences were considered significant at p<0.05 and highly significant at p<0.01.
